# Legal pluralism and the production of (un)certainty in lived migration orders

**DOI:** 10.1080/27706869.2024.2366070

**Published:** 2024-07-22

**Authors:** Larissa Vetters, Carolien Jacobs, Sophie Andreetta

**Affiliations:** a Law & Anthropology Department, Max Planck Institute for Social Anthropology, Halle, Germany; b Van Vollenhoven Institute for Law, Governance and Society, Leiden University, Leiden, The Netherlands; c Laboratoire d’Anthropologie Sociale et Culturelle, University of Liège, Liège, Belgium

**Keywords:** Legal pluralism, uncertainty, social security, migration, relational state

## Abstract

This article builds on three of Keebet von Benda-Beckmann’s core concepts, namely, legal pluralism, social security and relational social theory, to reflect on the place of law in lived migration orders, both in the global north and in the global south. To do this, we build on three empirical case studies from our respective fieldsites in Germany, Belgium and the DRC. These cases illustrate how ‘thinking with Keebet’s work’ not only offers useful ‘sensitizing concepts’ for the empirical study of migration law and migration studies more broadly, but also provides a much-needed conceptual vocabulary to speak to and intervene in current debates in more doctrinal legal scholarship on global migration law. With this double aim in mind, we first build on a legal pluralism perspective to show how migration governance can be better understood through the prism of ‘lived migration orders’. We then suggest rethinking state sovereignty, citizenship, and individual human rights in light of historical and newly emerging relational configurations. Finally, we suggest a new avenue for reconceptualizing migration governance beyond rights-based categorizations of migrants by paying more nuanced analytical attention to situations of uncertainty and how they contribute to social (in-)security.

## Introduction

Legal pluralism was the major focus in most of Keebet’s work throughout her rich academic career. The concept was close to the heart of both Franz and Keebet, and they published widely on it (Benda-Beckmann 2001; Benda-Beckmann 2002; Benda-Beckmann and Turner 2020). Another focus of Keebet’s work was social security in South East Asia and in other ‘developing countries’. She worked on this on her own, for example in her research with Moluccan migrants in the Netherlands (Benda-Beckmann 2015), as well as with Franz and other collaboration partners, such as the research group on social security and the local state in rural communities in former Eastern Europe, in which she was active during her last years before retirement (Thelen and Pasieka 2012). In these years, she became increasingly interested in looking at social security, legal pluralism and the state through the lens of relational social theories (Thelen, Vetters, Benda-Beckmann 2018; Benda-Beckman 2021). Although Keebet frequently linked these discussions to the topic of migration, she did so primarily as a means of illustrating certain aspects of legal pluralism, social security or relational personhood and intergenerational conflict. Her research on the lived experience of migration, however, engages less with the legal pluralism ingrained in migration orders. Our contribution proposes to further connect legal pluralism, social security, and relational theory in a more encompassing conceptual framework for the doctrinal and sociolegal study of migration.

Global migration governance consists of a patchwork of rapidly changing rules and regulations that often have limited temporal validity. Migration law is situated at the intersection of domestic sovereignty and international regulation, and oscillates between the contradictory objectives of human rights protection and migration control. It has developed incrementally and in a piecemeal fashion, often addressing and categorizing migrants according to the frequently shifting priorities of receiving states. This has led to increasingly complex differentiations of migrants according to motivations (voluntary or forced), grounds of admission (asylum, labour/education, family reunification), legal status (legal title, tolerated, undocumented), and duration of stay (transit, temporary, long-term). Different categories of migrants are governed by distinct regulatory frameworks (Chetail 2019, 6-7). These frameworks and their underlying logics are currently coming under stress as they are criticized on normative as well as empirical grounds (Achiume 2019; Byrne, Noll, and Vedsted-Hansen 2020; Kihato and Bakewell 2022; Bakewell 2008; Crawley and Skleparis 2018).

In this article, we take the concern for rigorous and precise use of analytical concepts coupled with ethnographic detail that animated Keebet’s scholarly work as a stepping stone to contribute to current efforts to rethink migration law and governance. As scholars with backgrounds in legal anthropology, public administration and development studies, we take a grounded and actor-oriented perspective, starting from the analysis of our own empirical data. We connect empirical realities with more normatively oriented understandings of legal pluralism and ongoing theoretical debates in migration studies. Migration studies is traditionally a multidisciplinary field, yet the production and dissemination of knowledge is often siloed (Arar and FitzGerald 2023). In our discussion we aim to abandon these silos by consciously not restricting ourselves to a particular type of migration, by combining data from fieldsites that may have little in common at first glance, and by combining theoretical concepts from different disciplines.

We proceed as follows: In the first part, we summarize and outline our understanding of key analytical concepts central to Keebet’s work – legal pluralism, social security and uncertainty, and a relational social theory. The second part consists of three ethnographic descriptions in which migrants, state officials and other actors navigate and shape various normative orders to better realize migrants’ fundamental rights. In the third part of the article, we draw on these three cases to illustrate how ‘thinking with Keebet’s work’ can connect to and enrich current debates in both doctrinal and empirical migration law scholarship and migration studies more broadly. We suggest three analytical moves. First, we propose to understand and analyse migration governance through the prism of ‘lived migration orders’ (building on a legal pluralism perspective). Second, we rethink state sovereignty, citizenship and individual human rights in light of historical and newly emerging relational configurations. Finally, we reconceptualize migration governance beyond needs- or rights-based categorizations of migrants by paying more nuanced analytical attention to how legally and socially produced (un)certainties are interlinked with material (in)securities and contribute to either reproducing or addressing structural inequalities.

## Building on Keebet’s work: a relational approach to understanding responses to uncertainty in lived migration orders

### From disputing to overcoming uncertainty and insecurity

Studies in the field of legal anthropology in general, and legal pluralism specifically, have traditionally had a focus on disputing, primarily centring on the navigation of legal pluralism by disputants often in colonial or early postcolonial contexts. It was widely assumed that disputes opened a window onto studying state and non-state norms, because norms tend to become more explicit at the moment they are contested. To the study of disputes Keebet contributed the concepts ‘forum shopping’ and ‘shopping forums’, emphasizing not only that disputants could strategically move between different forums to find justice or a resolution of their disputes, but also that the forums themselves, i.e. the intervening parties, could strategically invoke different sets of norms at different moments in time (Benda-Beckmann 1981).

Yet as early as 1973 Holleman’s work on trouble-cases and trouble-less cases showed that disputes may not reveal much about how a society functions under normal circumstances, and therefore made a plea to look into ‘trouble-less cases’ to more fully understand social order (Holleman 1973). This resonates with Franz and Keebet’s observations that disputes are not necessarily the natural counterpoint to social order and normality. In fact, they argued that ‘the more basic counterpoint to social order may not be conflict or disruption, but uncertainty and insecurity’ (Benda-Beckmann and Benda-Beckmann 2000, 7). They explained that

in each social organization there is a range of more or less satisfactory ways to deal with the material and immaterial aspects of uncertainty and insecurity in problematic life situations. Social relations and institutions and cultural or religious belief systems always, preventively or reactively, provide, or promise to provide, some help and assistance to those who are unable to help themselves. (ibid: 7)

It is here that we see the contours of both a social security and a legal pluralism perspective coming together in the work of Keebet. Social security, according to Keebet, can be conceptualized as social arrangements that go beyond formal institutional provisions and include the combined efforts of individuals, groups, and organizations in interlinked national and transnational social fields to overcome insecurities (Benda-Beckmann 1991, 2004; Benda-Beckmann and Benda-Beckmann 2000). Following Keebet, we approach social security as ‘a short-hand term for the social responses of support to situations in which persons cannot take care of themselves or threaten to lose the ability to provide for themselves’ (Benda-Beckmann 2004: 3). We suggest extending this understanding of social security as a multi-faceted relational response to the uncertainty that often characterizes migration governance. Stepping away from the idea that migration governance oscillates between contradictory objectives of human rights protection, on the one hand, and a state’s sovereign right to control migration on the other, we explore what it would mean to conceptualize migration governance as a response in the form of social support in situations where persons may lose the ability to provide for themselves. To this end, we expand on Keebet’s concept of social security by exploring different dimensions of existential material, economic and social insecurity as experienced by migrants, and asking how these insecurities articulate with different forms of legal and social uncertainty that migrants are facing when trying to (re)build secure lives in a new place of residence.

In this understanding, social security is a response not only to legal and social uncertainty, but to existential material insecurity as well. It emerges through diverse practices, relationships, ideologies, policies, and institutions that are not necessarily contained within the boundaries of a nation-state and its administrative apparatuses, but – as relational configurations – can also extend beyond the nation-state. Some individuals partaking in these relational configurations and their emerging normativities of care, we argue, can also represent ‘the state’ and contribute to security from this position (see Benda-Beckmann and Benda-Beckmann 1998).

### Towards a relational approach

We find a relational approach particularly helpful for better understanding how people navigate and seek to overcome uncertainty and material insecurity by relating to state and non-state actors, as well as to formal legal frameworks and informal norms. In this process people shape, reshape and bend the rules of the game. In a string of publications, Keebet emphasized the relational aspects of personhood, the state, and legal pluralism with a particular focus on social security arrangements (see, e.g. Benda-Beckmann 2015; Thelen, Vetters, & Benda-Beckmann 2018; Benda-Beckmann 2021). Challenging the common liberal assumption that an individual is essentially separate from others, which forms the bedrock of much legal thinking, Keebet was able to revisit how rights and duties of support are distributed in social relationships and settings in which a variety of normative orders are at play. Since people depend on the support of other individuals, collectives or state institutions for social security, establishing significant relationships is of vital importance. Not only the self, but also the respective significant counterparts (other individuals, family, religious and other collectives, state actors, etc.) are mutually constituted within these relations. In the processes of negotiating rights and duties of support, notions of personhood (both legal and social) as well as the meaning of kinship, state, and law are shaped and reshaped in multiplex relational configurations (Benda-Beckmann 2015; for further examples, see Thelen 2015; Thelen et al. 2018; Drotbohm 2020).

Within the abovementioned research group on social security and the local state in Eastern Europe, this relational approach was further developed with a specific interest in relational modes of engaging with the state (Thelen, Vetters, & Benda-Beckmann 2018). The authors describe the state as a relational setting that cannot be categorized according to simple hierarchies of governance (such as in a federal system) or by a monolithic governing apparatus (such as in centralized states). Rather, the state is seen to exist within the relations between actors who have unequal access to resources (material, social, regulatory, and symbolic). By drawing on existing images of the state, these actors negotiate ideas such as state sovereignty, the state’s authority to impose binding rules or to legitimize its power, and expectations of state support. In doing so, they at once reaffirm and transform these representations within concrete relationships that draw on and incorporate a multiplicity of normative orders. In one of her last articles, Benda-Beckmann (2021) adds a stronger emphasis on legal pluralism to analyse the connections between care/social security (within and beyond the welfare state) and the relational study of statehood. Drawing on examples from Indonesia, Thailand and the Netherlands, she traces how normative orders change over time, incorporate changing conceptions of personhood in a piecemeal fashion and, at the same time, affect broader changes in societal notions of personhood. Formal legal personhood, with its attribution of rights and duties of support, does not, however, necessarily reflect the perceptions and practices of those at the receiving end of the law. This, she holds, constitutes an important source of uncertainty and discomfort. Keebet concludes that ‘a truly relational take on legal pluralism’ calls for a methodological recalibration that includes ‘a broader conceptualization of what constitutes a group, a semi-autonomous social field, or social world’ (2021: 17–18). Rather than envisioning a group, a semi-autonomous social field, or a social world as being premised on a shared understanding of norms, in relational configurations different norms overlap and affect each other, but they might also be only partially known and differently understood. A relational analysis therefore also ‘reveals the extent of ambivalence, incoherence, misunderstanding, and uneasiness in relationships, interactions, and negotiations under complex normative conditions’ (2021: 20). This insight is particularly relevant in the context of migration. When moving from one physical environment to another, migrants maintain some relationships transnationally, but also often need to reshape significant parts of their relational configurations and seek new connections and interactions. As such, their relational configurations cannot be understood as social fields based on a shared understanding of norms, but are characterized by partial knowledge, overlapping and sometimes conflicting norms, incoherence, ambivalence and uncertainty. Migrants navigate lived migration orders that can be seen as relationally shaped normative arrangements. Because they are lived and relational, these orders are always characterized by a degree of normative uncertainty, often making it difficult for actors within these orders to know the rules of the game.

## Addressing the lived experience of uncertainty and insecurity: three empirical case studies

In the following sections, we explore different ways in which migration orders are being shaped and reshaped by state and non-state actors, including migrants themselves, and how this either creates or helps to overcome uncertainty and insecurity for migrants. Our combined ethnographic case studies look at different categories of actors (judges, welfare bureaucrats, and migrants), different policy/legal fields (residence permits, social assistance, and work) and different geographical contexts (Germany, Belgium, and the Democratic Republic of Congo) to analyse where, when and what types of uncertainty and insecurity arise in these different fields, and how actors navigate them. Although these cases are drawn from very different settings, they all illustrate how actors situated in relational networks play with (and within) various normative orders (either competing or parallel) to create more security for themselves. These networks are partly actively created by migrants and partly they are embedded, by default, in existing networks.

### Navigating and shaping migration orders in Germany as a relational practice to overcome legal uncertainty and existential insecurity

Crossing state borders typically - or at least in principle - involves contact with the formal law of the receiving state. Two legally prescribed avenues for entering Germany exist: obtaining a visa and entering legally for a specific purpose, as defined in the visa (visit, education, employment, family reunification); or claiming asylum upon arrival in Germany and undergoing an asylum determination procedure. But in practice these seemingly separate and clear-cut legal frameworks intersect and are shaped by further norms originating in normative orders that may be local or translocal, formal and codified, or habitual and customary. This section draws on ethnographic data and secondary sources to describe such a constellation in which migrants’ and state actors’ interactions are shaped by complex legal frameworks and normative orders. It does so by examining the trajectories of rejected asylum seekers and their attempts to remain within the country, regularize their legal status, and (re)build a sustainable life.

Rejected asylum applicants who are still awaiting an appeal decision are assigned a preliminary residence permit (*Aufenthaltsgestattung*). Rejected applicants, who – for various reasons – cannot be returned to their country of origin even after a failed appeal, are granted a temporary suspension of deportation (*Duldung*). These short-term legal statuses limit their social rights and exclude them from many resources that would otherwise enable them to work towards self-sufficient lives,^1^ keeping them in a position of material dependency on support from state and non-state actors as well as in temporal state of protracted limbo and uncertainty. Achieving a legal status that offers a longer temporal perspective (i.e. greater ‘certainty’) and an enlarged bundle of social rights (‘security’) thus becomes a core objective in the quest for rebuilding a sustainable and self-sufficient life.

In the fall of 2016, at the administrative court of a major city in Germany, LV witnessed the asylum appeal hearing of a mother and her adult son from the Chechen Republic. They had applied for asylum in 2013. The mother’s application was rejected by the Federal Agency for Migration and Refugees (BAMF) in 2015 and the son’s in 2016, whereupon both submitted appeals in the city in which they had by that point lived for more than two years and where the son had meanwhile married and become a father. During the hearing at the administrative court, it became apparent how both the applicants and the presiding judge had engaged in a process of normative (re)construction and bricolage to find a solution for this family.

During the discussion of facts, Judge Maurer did not hide the fact that she was not fully convinced by the son’s and mother’s explanations about their persecution and flight. She also did not believe that the mother’s medical condition was serious enough to justify a temporary suspension of deportation, as requested in the legal claim submitted by their lawyer. She then asked mother and son if there was anything else they would like to add to their explanations. Looking directly at the judge rather than the interpreter sitting by her side, the mother launched into an emotional appeal:

Mother: We left our country for a serious reason, but now my son also has a family here and for this family I beg you to let us stay here [she begins to cry]. Their second child will be born in a few weeks, and we will have a certificate of paternity. If we have to leave, these children will have to grow up without a father…

Judge Mauer [interrupting]: This is the one thing you can be sure about, the family will not be separated. Either you will all remain here, or you will all leave. Where does the mother come from?

Mother: She comes from Ingushetia. She has also applied for asylum and we believe she has good chances. She has received a letter for a hearing…

Judge Mauer: Yes, I saw in the file that there was a customary Islamic marriage and a child. I could not determine the nationality, but I have already submitted a request to the central registry of foreigners to clarify the legal status of your son’s wife. So far, I have not received a reply. [Turning to the lawyer] Do you know anything? [The lawyer shakes his head.] I will inquire again before I write the decision.

In hindsight, it appears that Judge Maurer had already anticipated that the solution for this family might not lie in the field of asylum law; their prospects for remaining in Germany might be better based on the right to family life as protected in general immigration law, and she proactively tried to further explore this option. As described elsewhere (Vetters 2022), such a proactive stance among judges towards ‘switching tracks’ from asylum to immigration law can be interpreted as part of an evaluative regime among judges consisting of sentiments and practices of procedural justice. This practical normative regime influences and structures their interactions with claimants in the oral asylum appeal hearing.

In this particular oral hearing, we catch glimpses of several normative orders that are partially mobilized and brought into the interactions by all actors involved. Both the migrant and the authorities ‘shop’ between different norms in their search for an optimal outcome (see Benda-Beckmann 1981). First, we encounter the doctrinal division between a humanitarian right to reside in Germany based on the Asylum Law on the one hand and, on the other hand, a right to reside in the country for other reasons (in this case, based on the right to family and private life protected under art. 8 ECHR), as regulated by the Residence Act. This normative order becomes porous in practice and is complemented with a situated and practical evaluative and normative register in which an asylum claim is transformed into a claim to family and private life. This normative register, in turn, is partially fuelled by European human rights law, which, through the jurisprudence of European Courts on the right to family life, intersects with domestic German immigration law, creating a pluralized normative landscape with some openings for rights claims based on kinship relations.

In this case, kinship relations are based on a marriage with another third-country national and the birth of children in Germany. The marriage was not conducted under German family law, but under the normative orders of countries of origin, which are plural legal orders as well. The spouses come from the former Soviet socialist autonomous republic of Checheno-Ingush, which was divided into the Chechen Republic and the Republic of Ingushetia in 1991–92. Both republics are today part of the Russian Federation, but experienced violent conflicts in the aftermath of the dissolution of the Soviet Union. Chechens and Ingush are predominantly Muslim. The co-existence and intermingling of Soviet/Russian state law, religious law (sharia) and customary norms (*adat*) is well documented for both republics (Adensamer 2012; Lazarev 2019). According to Adensamer (2012, 60), in this context the socially recognized and acceptable form of marriage is a religious marriage with a mullah. A civil marriage and formal registration can happen later, but is not necessary for recognition within the community.

Research by Sadyrbek (forthcoming) on the diasporic Caucasian community in Germany shows that a customary religious marriage is perceived to be of primary importance for granting legitimacy to the marriage in the eyes of this migrant community. However, at least two other factors have an impact. Even if couples wish to conclude a civil marriage according to German family law, they frequently will not be able to do so because they are unable to provide the necessary documents to the civil registry office.^2^ In addition, people may fear that procuring such documents from their home country could facilitate deportation if their asylum claim gets rejected. On the other hand, exclusionary migration laws provide an incentive to form a family in order to obtain a residence title through the legal protection offered to the unity of family life. What is legally relevant for the purpose of a residence title is proof of parenthood of children born in Germany whose parents are third-country nationals but are now based in Germany. Thus, it is not that the immigration laws encourage marriage as such; rather, it is the awareness of these indirect effects of establishing a family that makes customary marriage an attractive choice.

By means of customary marriage, normative expectations within the community are fulfilled, problems related to the lack or impossibility of a civil marriage are partially alleviated, and an avenue is opened to mobilize particular stipulations of migration law to avert a pending deportation and obtain another residence title.^3^ Even though such a residence title will also be temporary and will require further efforts to renew, it constitutes the first step in consolidating a more secure legal status that comes with more rights: employment restrictions are lifted; social assistance can eventually be granted according to the general social assistance law; and language and integration courses can become accessible. This status also entails a longer temporal perspective.^4^

By navigating and mobilizing plural legal orders, this newly formed migrant family is attempting to build a secure future. Kinship relations play a crucial role in this response to legal and social uncertainty. The original dyad consisting of a mother and her son who had migrated together is extended in Germany through marriage and the birth of children. This can be seen as an attempt to ‘inhabit’ a relevant legal category, and it can be successful because the underlying ideas about what constitutes a family and the obligations and rights attached to these kind of bonds (through marriage, but also and perhaps more importantly through descent) are similar enough to be translated across normative orders. At a later stage, intergenerational care relations within this extended family might also become legally relevant when it comes to the mother’s right to reside in Germany. Should she become dependent on care and if such care cannot be provided by relatives or state institutions in the country of origin, the actual care provided by family members in Germany can be recognized by current law as a legally significant social relationship of support that translates into a right to residence.

### Granting social assistance to the ‘unwanted’: illegalized migrants, welfare institutions, and productive uncertainty in French-speaking Belgium

Aside from their residence status, transnational migrants’ struggles for security also involve securing more specific social rights – and therefore, also, access to public welfare services. These include, for example, public shelter for asylum seekers, access to health care and, in some situations, social security benefits. This second case explores how public servants assess migrants’ social security claims in Belgium.

‘Laws are like a frame – we can put a lot of different things inside them, depending on state policies, internal guidelines, and the discretion of the person at the desk’, Paul, a social worker with the Public Center for Social Welfare (PCSW), once explained (interview, March 2019). Using welfare administrations as an example, this section explores the various, sometimes competing regulatory logics within state law that come into play when precarious migrants interact with street-level bureaucrats in their country of immigration. Drawing on ethnographic data, it shows how various layers of national and international laws create legal uncertainty for both state agents and migrants interacting with them. Such uncertainty, however, does not necessarily lead to ‘fewer’ substantive rights for claimants: unlike most migration bureaucrats, whose discretionary practices tend to compound scrutiny (Spire 2008), welfare bureaucrats sometimes make use of these uncertainties to try to help migrants get more (social) security.

In addition to insurance-based benefits, which are only accessible to those who have contributed to the specific social protection scheme that they relate to, Belgian residents can also access public social assistance, which is potentially available to all. Whether in the form of a monetary payment or of material support – such as direct provision of health care, medication, food, or energy – social assistance is meant to guarantee that people ‘live in conditions that conform to human dignity’ (Const. art. 23). While all forms of social assistance used to be accessible to all residents, in 1996, welfare reforms limited illegalized migrants’ social assistance rights to ‘emergency medical assistance’ (EMA), meaning they could only access public health care – provided that such care was deemed ‘necessary’ by a medical professional and according to administrative guidelines. A few years later, reception benefits for asylum seekers were also limited: first, material assistance within public housing centres replaced financial aid; second, assistance was further restricted to health care for those introducing a subsequent asylum request based on new facts. Against this backdrop, exceptions started to emerge in the case law: both national and international courts recognized severely ill migrants’ right to financial assistance while they were waiting for their immigration cases to be settled. Under the same logic, undeportable migrants, such as the parents of underage Belgian children, were also granted access to a minimum income. Within asylum reception centres, exceptions could be made for ‘vulnerable’ claimants.

In practice, these categories of rights are granted by two kinds of administrations: PCSWs and the Federal Agency for the Reception of Asylum Seekers. In both institutions, those directly in charge of interacting with migrants are, in most cases, trained social workers. Most social workers experience a degree of dissonance that comes from that fact: they highlight the contradictions between their professional values and normative orientation, centred on their desire to help, and the internal guidelines of the administration, which are frequently informed by a logic of migration control and deterrence. These contradictions are not limited to the Belgian case: welfare workers in other contexts, especially those dealing with migrants, have been described as ‘bound between care and control’ (Perna 2019; see also Achermann, Borrelli, and Pfirter 2023). This has also been referred to as the ‘double mandate’ (Schulte 2009) in social work. The Belgian case helps us go beyond the contradictions inherent in such a double mandate by showing how, in some cases, welfare workers can make use of the various layers of official norms to care for migrants.

Welfare administrations are responsible for implementing welfare laws; however, in practice, they are bound by administrative guidelines from the Ministry of Social Integration – which provides most of their funding. These guidelines are meant to provide interpretations of legal principles: they state which medical treatments are covered under EMA, the information needed for a claim to be assessed and for assistance to be granted, and what kind of benefits can be accessed by each category of residents. Every year, a sample of case files gets inspected and evaluated: if some of them reveal that assistance was granted in cases that did not fully comply with these guidelines, the responsible office’s funding can be reduced by an amount determined by the proportion of ‘incorrect’ cases. Because they create additional layers of rules and restrictions, administrative guidelines often end up not only limiting social assistance rights in practice (Andreetta 2022a), but also creating additional checks to be performed by welfare workers on the ground (Thiemann 2016: 161). A large part of their job indeed consists of verifying the eligibility of their ‘clients’ through two main information gathering techniques: the initial interview, where the social worker asks a series of questions regarding the persons’ administrative, social and medical situation; and a house visit, through which residency can be proven.

Administrative guidelines are, however, not legally binding: judges can overrule them if they feel that they provide incorrect or overly restrictive interpretations of welfare laws. Moreover, administrative or internal guidelines sometimes directly contradict judicial decisions, meaning that, when dealing with cases, welfare workers are confronted with several, sometimes competing sources of law. During the inquiry, a migrant is tied to a specific social worker who, in turn, is tied to the judge in a different relational configuration, mainly through the exchange of documents (Andreetta 2019).

Once the information gathering stage has been completed, social workers write reports and submit recommendations to the welfare office’s board. Most of them acknowledge that the way they describe a given situation, the information that is included or left out, and how the case is presented can easily sway the decision one way or another:

We have tremendous discretion in writing our reports. You can describe a case in a way that justifies the person’s situation and their choices, or you can cast suspicion on them as easily as snapping your fingers. (Eloise, interview, 2019)

Much like welfare investigators in France (Dubois 2014), social workers’ reports are meant to evaluate the consistency and credibility of an applicant’s story, on the basis of which welfare entitlements are determined. In such a context, whether welfare workers truly believe applicants or not can determine if they make their administrative journey harder or go the extra mile to help the applicant. While the Belgian case presented here helps unpack the procedural mechanisms that allow for the latter to happen, there are also many examples of welfare workers simply following administrative guidelines – much as Ticktin (2011: 104) described some humanitarian workers as being’ there simply as professionals, to earn a living’ – or using their discretion to deny migrants’ ‘incredible’ claims. Social reports therefore present a specific narrative (Ticktin 2011) in which the applicants’ deservingness is discussed (Andreetta 2022b). Social workers can argue against administrative guidelines, but the benefits granted would then have to be paid for from the office’s own funds. Such recommendations from social workers are, therefore, often disregarded by administrative boards. ‘Every year, I recommend that we grant them financial assistance’, Marie explains about a family without a legal immigration status. ‘But thus far, my recommendations have never been followed. But I think my job is still to say that they need help’ (fieldnotes, 2019). Nevertheless, recommending against administrative guidelines can still prove useful to the applicants: social reports are included as evidence and are read by the judge if a suit is brought against the administration. Writing ‘in favour of the user’ therefore constitutes one of the strategies that social workers use to support the granting of assistance despite the administrative guidelines and without infringing on them (Andreetta, 2022c). Talking about one such case, Francine explains:

I had a woman from Russia with two little girls. I sent her to court because one of her daughters had a rare disease and she needed a very expensive treatment – three 100,000-euro injections. Some researchers here asked her to come, so she quit her job as an economics professor, sold her house, everything, and she came. But they were not told how much the treatment was, and they couldn’t afford it. She had insurance, but it didn’t cover experimental drugs. I had to turn down her request because, even though she was not a resident here, she still had insurance. But I told her to go to the doctor and file a 9ter regularization^5^ request for her daughter. Now her case is being examined by the Aliens office. (Interview, May 2018)

While not all social workers know that the courts can challenge administrative guidelines, those like Francine who are used to assessing requests from irregular migrants are often aware that such a possibility exists. When they put forth a request that they know will likely be turned down by their own administration, they are playing a longer-term game: they are provinding claimants with a refusal of assistance from their office, thereby giving them the opportunity to take their case to court where their request might be granted. Like legal advisers in the UK (Forbess and James 2014), by writing reports describing applicants’ situations, welfare bureaucrats are building evidence supporting migrants’ claims.

In court, judges rely on welfare law principles, and must decide whether human dignity would be infringed on if social assistance were to be denied. Particularly on issues involving migrants, diverging trends co-exist within the jurisprudence: some regularly grant irregular migrants’ requests based on international law principles and the fundamental right to human dignity; others adopt a more restrictive approach based on national immigration and welfare law principles.

This example shows how legal uncertainty can work in parallel ways: welfare administrations, courts, and migrants must navigate competing, sometimes contradictory sets of norms. While this is of course harmful to litigants’ sense of legal certainty and often has material consequences – such as being denied housing, medical treatment, and other benefits – the dissenting voices both within and across state institutions also allow for contestations, debates, and, sometimes, for furthering illegalized migrants’ rights to social protection – as granted by the state – and therefore, to more (social) security.

### Joining forces to address uncertainty and to promote social security for internal migrants in the DRC

Whereas the previous cases shed light on transnational migrants’ efforts to rebuild their lives by achieving a certain level of legal and social security, the following example from the Democratic Republic of Congo (DRC) shows how internal migrants make use of the uncertainty surrounding rules to actively reshape labour conditions and to strengthen their own position. As such, migrants exert their agency and constitute a source of normative ordering as well. This phenomenon is not necessarily limited to the field of migration orders, but can be observed in other fields as well.

In the eastern part of the DRC, conflicts have been rife for more than three decades, with varying levels of intensity and multiple, shifting centres of insecurity. Displacement has become a recurring experience for many people. Moves are sometimes triggered by the immediate threat of violence; in other cases, a more general sense of insecurity is one of several factors that contribute to the decision to move. In practice, there is not a clear line between the internally displaced persons (IDP) category and other in-country migrants. Many people move for a combination of reasons, and these reasons often cannot be fully disentangled. Especially in the first months or years of displacement, many people have limited social connections to other citizens or to the local state authorities, which limits their ability to navigate their new social surroundings.

At the national level, there is no specific legal protection framework in place for IDPs, although the DRC has signed the 2006 Great Lakes Protocol on the Protection and Assistance to Internally Displaced Persons. This protocol is legally binding and is supposed to be complemented with national legislation. In 2022, the DRC also finally ratified the African Union Convention on the Protection and Assistance for Internally Displaced Persons in Africa (the so-called Kampala Convention).^6^ In practice, IDPs – especially those residing in host communities and not in camps – receive almost no targeted protections or social assistance through formal channels.

Outside the purview of the state authorities, IDPs and other migrants stick to their familial or ethnic networks to navigate their place of refuge, to seek integration, to realize their fundamental rights, and to achieve a sense of security. Ties that are created through these networks can be sources of new normative orders and can help newcomers strengthen their position vis-à-vis others. Many labour branches have organized themselves into associations in which members unite to negotiate their working conditions.^7^

A case in point is an association of displaced vulnerable women in one of the eastern provinces. Without external support, this group of about 80 women has organized themselves not only as a social safety net and as a savings and loan association, but also as a labour union to jointly negotiate work with their contractors in an urban setting. The following case sheds light on this association and how it helps displaced women negotiate their labour rights and achieve a certain level of social security. It demonstrates that migrants are not only adopting and responding to formal and informal norms with which they are faced in their new environment, but also how they take steps to shape these norms and, as such, change the set-up of the host society in an effort to realize their rights.

In one case, the women jointly carried out a major task for a contractor on the shores of Lake Kivu, for which they had been paid $200 in advance and would receive $300 upon completing the work. With a group of about 50 women they managed to finish the work in two weeks’ time, whereas the expectation had been that the work would take four weeks. Instead of paying the outstanding $300, the manager of the contracting enterprise gave them new tasks to fill the full four weeks. The women refused to execute these tasks, asserting that they had fulfilled their contractual obligations by completing the initial task. The impasse had already lasted for three weeks when the women decided to file a complaint with the naval commander of the Congolese Armed Forces. One of the women explained:

We went to seek mediation from the commander of the FARDC [Congolese Armed Forces] marine section units because they are managers of lake areas and also managers of disputes and misunderstandings that arise in connection with lake operations. (Group interview, July 2015)^8^

The commander did not manage to reach an agreement during his mediation efforts and ordered that all activities along the lakeshore be suspended, which then drew the attention of the head of the enterprise. The head indicated that he had paid the full sum to the manager at the start of the task, implying that the manager should be held accountable. Nevertheless, the head of the company paid the outstanding money to the women and suspended his manager for six months.

The involvement of the commander helped the women shape the rules of the game with the manager, who had expected he could get away with applying his own rules. The result was obviously positive for the women and secured their rights. In addition, from then on, the commander of the marine unit decided that all agreements for the execution of work along the lakeshore should be submitted to his office for approval to increase the security of the workers. Reflecting on the newly instituted norms, one of the women said:

From our side this measure is good, because we were already overwhelmed by similar cases and often got threatened by other people executing the same work as we do. They blamed us, saying that we accept very low pay because we are displaced and do not know all the socio-economic realities of the city very well. (Group interview, July 2015)

The hidden drawback, however, was that for all agreements for the execution of work on the lakeshore, the commander charges 5 per cent of the agreed payment for overseeing the work.

We talked again to some of the women some weeks later, by which time they no longer worked at the lakeshore, but only carried out smaller jobs in the neighbourhood because they felt the 5 per cent charge was too high. They had noticed that fewer people worked at the lakeshore because the conditions had become less attractive. A further complexity arose when the commander and his troops were replaced and no one knew the new commander, underlining the women’s reliance on maintaining good personal connections with authorities to be able to secure their rights.

Through joint negotiations, the displaced women of the association strengthened their position vis-à-vis other actors and reshaped some of the rules of the game. Such joint action underlines how migrants not only follow the normative orders in their host society, but also actively reshape some of the norms to realize a stronger and more secure position. The staff turnover in the armed forces, however, made them feel insecure again, underlining the need for a stable institutional environment in which people can rely on the continued application and acceptance of rules. The resulting uncertainty reduced the displaced women’s sense of security.

Refugees and other cross-border migrants often have many hurdles to overcome to be able to claim their ‘right to have rights’ and to secure their place in a new country. IDPs and other internal migrants are citizens within their own country and should be able to find security in their communities of settlement. But when such a host community is characterized by informality, even within formal state institutions, it is not always easy to navigate these norms, as the authorities may also randomly and unexpectedly go ‘shopping’ for different norms. The case from the DRC shows how crafting relationships with other migrants and with authorities can help in reconstituting the norms that are in place to overcome uncertainty and to realize greater security.

Whereas IDPs often benefit less from particular protection orders than do refugees, they are also not hindered by the mobility restrictions that are imposed by these orders, as they are citizens within their own country. At the same time, they constitute a category of people who do not belong and who face a degree of existential uncertainty. For them, as for most conflict-induced displaced people, uncertainty – rather than certainty – is the norm (Horst and Grabska 2015). Shared origin, a shared migration experience, and a shared need for more certainty constitute the grounds on which the women – seemingly without much power – formed their association to improve their labour conditions and reshape the labour norms in the harbour.

## Legal pluralism, relational configurations, and (un)certainty/(in)security in the study of lived migration orders

What do our combined cases show when analysed through the lens of Keebet’s conceptual work? And how does this complement ethnographic studies and arguments made by other migration scholars (anthropologists, sociolegal scholars and doctrinal legal scholars alike)? First, we apply legal pluralism as a sensitizing analytical concept for our case studies (Benda-Beckmann 2002: 40; Benda-Beckmann and Turner 2020; Shahar and Yefet 2023). This allows us to adopt a more symmetrical approach to studying migration orders. Read together, our cases indeed demonstrate that not only do migrants – be it in the DRC or in Germany – shape and reshape the (formal and informal/practical) rules of the game when they navigate multiple normative orders in their new place of residence, but that state actors – such as welfare bureaucrats in Belgium and administrative judges in Germany – also navigate the incoherence and gaps that exist in the interstices between different regulatory frameworks. In Keebet’s words, migrants go shopping among different forums to find the one that works best for them in a given situation, and forums themselves shop among different norms (1981). In the process, all of these actors continuously contribute to the incremental and piecemeal transformation of norms.

Second, our cases also allow us to take a more encompassing view of migration governance – for which we suggest the alternative concept of ‘lived migration order’. Lived migration order denotes the empirically traceable, situated assemblage of normative elements that emerges in specific encounters and interactions between migrants, those tasked with enforcing black-letter law, and other societal actors. Because of their situatedness, such migration orders are often fluid and are shaped by the way in which different actors selectively engage with, ignore, or reshape norms. Sometimes this results in relatively stable configurations; in other instances, such configurations remain dynamic, which makes it difficult to predict the course of events. In all three of the cases presented here, migration orders can be conceptualized as inherently evolving and open to reconfiguration.

Finally, in both doctrinal and sociolegal scholarship, uncertainty is often highlighted, but rarely unpacked. Reading our cases through the lens of Keebet’s work allows for such an unpacking of uncertainty in its interdependence with material (in)security and the reproduction or alleviation of structural inequality. In the following sections we further expand on Keebet’s conceptual thinking and the larger field of migration studies in relation to our empirical findings.

### Legal pluralism as a sensitizing concept for a symmetrical approach to lived migration orders

To date, the governance of migration has often been studied either through state practices (Spire 2008; Fassin 2010; Eule, Loher, and Wyss 2018; Infantino and Sredanovic 2022) or through the informal strategies of migrants themselves (Le Courant 2016; Psimmenos and Kassimati 2006; Vasta 2011). In these approaches, bureaucratic and judicial discretion is often described as happening outside of regulatory frameworks, at the margins of the rules, or in the discretionary spaces that emerge through street-level practices (Spire 2008; Le Courant 2016; Soennecken 2016). Migrants’ strategies, for their part, are frequently conceptualized as extra-legal responses to formal state law or as rights-claiming practices outside the framework of legally recognized rights (Le Courant 2016; Psimmenos and Kassimati 2006).

A small and rather diffuse body of recent literature has aimed to overcome this dichotomy by studying specific lived migration orders within a single overarching analytical framework based on legal pluralism. Among these studies are Glick-Schiller (2005) and Barbero (2013), who focus their analyses on the multiple normative orders that migrants engage with and help to reshape by claiming rights that are formally denied to them, and Moffette (2020, 2021), who directs attention to the ‘interlegal jurisdictional games’ that state actors play when they cherry-pick legal tools to govern, control and punish immigrants. As we will show below, these studies of ‘lived migration orders’ in the European context should be read together with similar studies from the Global South. Here, McConnachie (2014) shows how refugees, despite being dependent upon their host states and international organizations, nevertheless exert individual and collective agency when they (re)build forms of community governance using a combination of spiritual beliefs, traditions and customs, moral didacticism, and various forms of codified national and international law. Landau (2014) forcefully argues against the categorization of IDPs as a separate policy category governed by a distinct legal framework. He proposes ‘a substantial redefinition of the modes through which we “see” and understand displacement’ in urban settings in the Global South (ibid: 140). Instead of maintaining the usual categorial distinctions applied in policymaking, such as the distinctions between emergency assistance and durable solutions, between formal and informal economies, and between IDPs and other vulnerable urban residents, policymaking should support the formation of cross-cutting social networks (ibid: 139, 146–7).

These studies provide further clues on how to apply legal pluralism as a sensitizing concept to our case studies: The German case study, read together with Glick-Schiller (2005) and Barbero (2013), powerfully demonstrates that empirically tracing migrants’ rights-claiming practices as a window into the mobilization of multiple normative orders is an important technique for elucidating lived migration orders.

Our German and Belgian cases also highlight state actors’ choices in applying certain norms and not others. Contrary to Moffette, some state actors in our case studies are sympathetic to the normative expectations of migrants for a sustainable life in dignity and are also influenced by their own professional ethics and values. Especially in the Belgian case do we see how social workers make use of discrepancies between legal sources and the dissenting voices both within and across state institutions. In line with Moffette’s call to take the broader sociolegal governance of immigrants into account, our cases thus demonstrate the usefulness of a broad understanding of lived migration orders, encompassing a variety of fields of state law and other normative orders, including welfare law, as in the case of Belgium, and labour law as in the case of DRC. Our perspective captures not only deterrence and exclusion (the punitive side of the state), but also takes into account the benevolent side of the state and pays particular attention to how restrictive and protective norms are interwoven.

Our DRC case is another example of a Global South context, in which migrants exercise their agency to influence the rules of the game in their new environment from a position of structural disadvantage. Moreover, the case study underlines that it is not only migration orders that impact migrants’ lives, but other orders as well, such as labour regimes. It demonstrates the limits of predetermined policy distinctions between IDPs on the one hand, and refugees on the other hand. Heeding Landau’s call to situate ‘displacement’ in context and adding a legal pluralism perspective makes it possible to bring lived migration orders into one analytical framework with other co-existing multiple normativities that shape (and are shaped) not only by migrants, but by all residents.

### Relational configurations and reshaping the rules of the game

Our three cases constitute a springboard for thinking beyond the dichotomy of state sovereignty to control migration on the one hand, and universal but individual human rights guarantees on the other hand – a dichotomy which runs through much of migration law scholarship. In all three cases, state sovereignty is decentred and replaced by a fragmented, non-unitary assemblage of state institutions and actors. Not only in the DRC, often associated with a weak state and fragmented institutional landscape, but also in Germany and Belgium do state representatives enact (migration) laws in distinctive and sometimes contradictory ways, sometimes even integrating nonstate norms. Moreover, instead of individuals claiming rights, we are more likely to see social collectives such as family units and women’s associations mobilizing a plurality of norms. What connects these two observations is a relational approach that allows us to empirically trace how configurations of statehood and (non-)citizenship are enacted in webs of relationships and personal or mediated interactions. Within the field of empirical migration studies, relational approaches exist and address important questions of structural inequality in relation to migrants’ and state actors’ agency, but they often do so from distinct perspectives. Building on a figurational perspective (Elias 1978), Etzold et al. (2019: 2) analyse protracted displacement as ‘dynamic social constellations between interdependent individuals that are produced in and through interactions and transactions’. Acknowledging the structural factors that constrain the agency of displaced persons, they nevertheless argue for recognizing displaced persons’ capacities and practices to form local and translocal networks in their quest for livelihood security. Focusing on relational decision-making among state actors responsible for welfare and migration control in Switzerland, Achermann, Borrelli, and Pfirter (2023), on the other hand, highlight how this relationality contributes to the co-production of bureaucratic legitimacy for decisions to deport ‘poor others’ and invisibilizes structural inequalities. Such empirically grounded relational perspectives have, however, gained relatively little traction in doctrinal debates.

We see our proposal for an empirically grounded relational approach to studying lived migration orders as also speaking to and furthering doctrinal debates in migration law that more fully address questions of historical and present-day structurally embedded inequalities. First, such an approach would appear to articulate well with recent doctrinal suggestions to rethink the autonomous individual legal subject through the lens of vulnerability, that is, as an embodied and relationally embedded legal subject in need of substantive rather than formal equality (Fineman 2008, 2024). This notion of vulnerability has been taken up by several migration law scholars, some of whom have criticized current uses of the concept that make of it yet another bureaucratic category for assessing needs and distributing assistance within traditional frameworks of migration governance (Leboeuf 2021; see also Andreetta and Nakueira 2022). To counter this tendency, they emphasize the need to take into account empirical findings about situated and contingent experiences of vulnerability (Baumgärtel 2020; Küçüksu 2022). Some of these authors seek to build a more encompassing theory of rights, based on universal, but situated and context-specific, vulnerability. At the core of their reasoning is the question of whether there can be a legal theory that addresses the needs of migrants without relying on and reaffirming an underlying division between citizens and noncitizens (Da Lomba and Vermeylen 2023) or, alternatively, one that provides legal mechanisms to address structural inequalities that arise when assigning a legal status that carries with it inbuilt legal uncertainty and material insecurity (Baumgärtel and Ganty 2024). Our ethnographic cases are a reminder that such questions of legal theory and doctrine are always and inevitably already being negotiated in practical everyday encounters between differentially situated actors.

Second, an empirically grounded relational approach to lived migration orders, especially if it also incorporates historical analyses, could enrich normative debates around the decolonization of migration law (Achiume 2019). Looking at the governance of migration through a legal pluralism perspective that acknowledges lived migration orders and how they are shaped and changed through relational configurations over time can, we believe, shift doctrinal debates on migration law towards new grounds that are more attentive to uneven power relations between different actors in the local and global context.

### Rethinking uncertainty in lived migration orders and beyond

Our three empirical accounts have brought to light a range of manifestations of experienced, perceived, and acted-upon uncertainties and existential material insecurities that warrant further analytical and conceptual attention. These empirical manifestations go beyond contemporary scholarship on (forced) migration, much of which still assumes that uncertainty, to some degree, originates primarily in the event of forced displacement; that it is inherent in and particular to displacement situations; and that it is a negative, constraining condition and a radical and abnormal state (see Schiltz et al. 2019 for a critical overview). Uncertainty (in the sense of radical uncertainty in contexts of conflict and flight as well as protracted uncertainty in the context of exile) is undoubtedly a key feature of (forced) migration. Nevertheless, as pointed out by Horst and Grabska (2015), is not necessarily an exceptional state of human existence, and it is also often socially and legal produced in receiving countries as a deliberate strategy of migration governance (ibid.; Maas et al. 2021).

Finally, it also results from partially overlapping, partially conflicting normative orders that come into play in relational configurations of mutual support and belonging.

Scholars such as Menjívar (2006), Griffiths (2014) and Biehl (2015) have shown how migration law and its implementation have become sources of uncertainty in and of themselves, either through the proliferation of temporary legal statuses with graduated sets of rights or arising from the incoherence of interpretation and opaque discretion that characterize multi-actor, multi-level and rapidly changing regulatory frameworks. Our German and Belgian cases mirror these observations, but they also demonstrate that these socially produced states of uncertainty are not categorically linked to heightened or diminished states of material existential insecurity. While a legal status such as the German *Aufenthaltsgestattung* restricts the scope of social rights for migrants and produces material insecurity, the legal uncertainty arising from margins of interpretation, the overlap between European and domestic law, and changing jurisprudence can also be leveraged in relational negotiations over plural normativities, resulting in a somewhat more secure legal status with better access to social rights. And while ‘jurisdictional games’ can be used as a control-and-deterrence strategy by some state actors, other state actors, such as the social workers in our Belgian case, might subscribe more strongly to a normativity of care, using normative indeterminacy as a tool to further migrants’ access to social assistance, even though their formal rights to social assistance are limited as non-citizens.

In the case of the DRC, internal migrants are not exposed to the same kind of legally produced uncertainty as cross-border migrants, and their rights to state-provided assistance are not formally questioned. Rather, they face an environment in which they share economic precarity and a lack of state-provided social security and institutional predictability with large parts of their host population. In such a setting, the displaced participate in shaping the ‘rules of the game’ in relational configurations in which various norms are contested, negotiated, reinterpreted, and partially remade, but the game in question is not a particular migration order. It is a broader socio-normative order that encompasses all urban residents, who participate equally in its constant, practical (re)enactment. As such, the DRC case helps us to understand that uncertainty is not only an abnormal condition that applies particularly to forced migrants, but that it can extend to all urban residents. This, in turn, also opens an analytical window for thinking about legal uncertainty and social well-being beyond the frameworks and categorizations of migration governance, moving more towards a broader conceptualization of social security that encompasses newcomers and long-standing residents, citizens and non-citizens alike. One the one hand, such a move would connect well with those voices within migration studies that criticize migration scholarship’s unreflective use of legal and policy-oriented categorizations of migrants, pointing out the risks of such categorizations (Bakewell 2008; Landau, 2014; Crawley and Skleparis 2018; Menjivar 2023) and calling for a ‘decentring’ of migration studies (Purkayastha 2023; Nieswand and Drotbohm 2014; Diederich and Nieswand 2020).

On the other hand, unpacking uncertainty in all of its socially and legally produced guises as it interacts with the material insecurities of migrants and non-migrants alike allows us to go beyond the necessary critique of state- or policy-created categories and classification systems as maintaining and reproducing systemic inequality, and enables us to return to normative questions of migration governance from a different perspective. As Keebet von Benda-Beckmann reminded us, ‘a fundamental level of uncertainty is not necessarily an anomaly’ and ‘social life can be no more than partially determinate’ (2018: 82). How would we envision the governance of migration if we were to start from the premise that ‘organisations, including polities such as the state and its laws and regulations [everywhere], are never more than “islands of determinacy in a sea of indeterminacy”’ (ibid.: 82)? Would this allow us to better conceptualize migrants’ and non-migrants’ quest for existential security beyond the limits of methodological nationalism and by considering the normative plurality of lived migration orders?

## Conclusion: towards a relational approach to migration

In this paper, we have brought together findings from three different field sites to shed light on uncertainty within lived migration orders, informed by Keebet’s work on legal pluralism, social security, migration, and a relational social theory. This endeavour has allowed us to suggest potential new avenues for the study of migration governance, migration orders, and the ways in which migrants overcome uncertainty.

Our article turns the usual legal pluralism lens around and begins with state actors. Our first case shows how a German judge navigates immigration law and uses hearings to steer cases in a different – and more promising – direction regarding migrants’ residence applications. The second case unpacks Belgian welfare bureaucrats’ interactions with precarious migrants, analysing how both categories of actors make use of the inconsistencies and uncertainties inherent in welfare and immigration laws to grant migrants more social security. In the DRC, internal migrants do not need to worry about their legal status, but to realize some degree of social security, they actively seek connections with each other and with state officials to overcome the uncertainty that is related to the precarious labour conditions they often face and to formalize their relations with contractors. In the context of a more fragile state, actors purposefully try to create mechanisms of certainty. Based on these cases, we first discussed legal pluralism as a sensitizing concept that allows for a more symmetrical approach to studying the emergence of lived migration orders. We then suggested that the study of relational configurations is central to understanding how norms (that is, the rules of the game) are reshaped. And we conclude with some thoughts on how rethinking the complex interlinkages between legally and socially produced uncertainty and material insecurity in lived migration orders can help to better understand and address the role of migration law in reproducing structural inequalities, or, conversely, alleviating them.

These three analytical takes could move current debates beyond the migration control and enforcement vs. fundamental rights dichotomy. They could also help to incorporate the normative power of a wide range of informal practices – such as solidarity networks and practices of care – into our understanding of lived migration orders. Our approach could, finally, contribute to providing answers to some normative questions by helping to grasp how rights and obligations are negotiated in relational networks in which notions of belonging, citizenship, and state sovereignty are reconfigured. A sociolegal understanding of lived migration orders, for example, contributes to a better understanding of the meaning of law and normative orders for migrants themselves and for the governance of migration. It can contribute to core questions of normative political theory by clarifying how relations between lawmakers and those subjected to the law are constructed in practice, how the separation of powers that legitimizes public authority becomes reconfigured in plural normative settings, how the tension between human rights and unequally distributed citizenship rights affects migrants rights’ claims, and how the practical and relational construction of legal (un)certainty relates to the normative goal of the rule of law.

## Notes

1 For those in the appeal process with an *Aufenthaltsgestattung*, for example, these restrictions include a requirement to reside in collective shelters for up to 18 months; a ban on employment during residence in a collective shelter; and a prohibition against leaving the municipality to which they have been assigned. Only basic medical services and social assistance are provided, as regulated by the *Asylbewerberleistungsgesetz*, which sets lower standards than the general social assistance act.

2 These include identity papers and a certificate of marital status issued by the country of origin. Such difficulties have also been documented for other migrant communities, where state institutions in the country of origin are hostile, dysfunctional or inaccessible (see Ferreri 2022).

3 In this case a residence title on humanitarian grounds according to *AufenthG* § 25 par 5, since an order to leave the country would be in violation of the protection of marriage and family life.

4 After five years of holding a residence title on humanitarian grounds, one can become eligible for a permanent residence permit if additional requirements are met.

5 The article 9ter of the 1980 Immigration and Aliens Act allows severely ill migrants to apply for legal residency, if they can prove that they cannot receive the necessary treatment in their country of origin and that without receiving it, their condition would severely worsen, putting their life at risk.

6 See https://au.int/sites/default/files/treaties/36846-sl-AFRICAN_UNION_CONVENTION_FOR_THE_PROTECTION_AND_ASSISTANCE_OF_INTERNALLY_DISPLACED_PERSONS_IN_AFRICA_KAMPALA_CONVENTION_1.pdf, viewed on 08.12.2023.

7 For the strong ‘associational life’ in this region of the country, see Hilhorst and Bashwira (2015: 17).

8 We have described elsewhere that going to the army or police for dispute resolution is often a step people take at second instance only, after having first tried to find ‘an amicable agreement’ (Jacobs 2018).
